# Improved prediction of Canada lynx distribution through regional model transferability and data efficiency

**DOI:** 10.1002/ece3.7157

**Published:** 2021-01-24

**Authors:** Lucretia E. Olson, Nichole Bjornlie, Gary Hanvey, Joseph D. Holbrook, Jacob S. Ivan, Scott Jackson, Brian Kertson, Travis King, Michael Lucid, Dennis Murray, Robert Naney, John Rohrer, Arthur Scully, Daniel Thornton, Zachary Walker, John R. Squires

**Affiliations:** ^1^ Rocky Mountain Research Station United States Forest Service Missoula MT USA; ^2^ Wyoming Game and Fish Department Lander WY USA; ^3^ United States Department of Agriculture, Northern Region United States Forest Service Missoula MT USA; ^4^ Department of Zoology and Physiology Haub School of Environment and Natural Resources University of Wyoming Laramie WY USA; ^5^ Colorado Parks and Wildlife Fort Collins CO USA; ^6^ Washington Department of Fish and Wildlife Snoqualmie WA USA; ^7^ School of the Environment Washington State University Pullman WA USA; ^8^ Idaho Department of Fish and Game Coeur d'Alene ID USA; ^9^ Environmental and Life Sciences Biology Department Trent University Peterborough ON Canada; ^10^ United States Forest Service Okanogan‐Wenatchee National Forest Winthrop WA USA; ^11^ Present address: Selkirk Wildlife Science Sandpoint ID USA

**Keywords:** Canada lynx, generalizability, GPS telemetry data, local adaptation, *Lynx canadensis*, niche similarity, regional variation, sample size, species distribution model, transferability

## Abstract

The application of species distribution models (SDMs) to areas outside of where a model was created allows informed decisions across large spatial scales, yet transferability remains a challenge in ecological modeling. We examined how regional variation in animal‐environment relationships influenced model transferability for Canada lynx (*Lynx canadensis*), with an additional conservation aim of modeling lynx habitat across the northwestern United States. Simultaneously, we explored the effect of sample size from GPS data on SDM model performance and transferability. We used data from three geographically distinct Canada lynx populations in Washington (*n* = 17 individuals), Montana (*n* = 66), and Wyoming (*n* = 10) from 1996 to 2015. We assessed regional variation in lynx‐environment relationships between these three populations using principal components analysis (PCA). We used ensemble modeling to develop SDMs for each population and all populations combined and assessed model prediction and transferability for each model scenario using withheld data and an extensive independent dataset (*n* = 650). Finally, we examined GPS data efficiency by testing models created with sample sizes of 5%–100% of the original datasets. PCA results indicated some differences in environmental characteristics between populations; models created from individual populations showed differential transferability based on the populations' similarity in PCA space. Despite population differences, a single model created from all populations performed as well, or better, than each individual population. Model performance was mostly insensitive to GPS sample size, with a plateau in predictive ability reached at ~30% of the total GPS dataset when initial sample size was large. Based on these results, we generated well‐validated spatial predictions of Canada lynx distribution across a large portion of the species' southern range, with precipitation and temperature the primary environmental predictors in the model. We also demonstrated substantial redundancy in our large GPS dataset, with predictive performance insensitive to sample sizes above 30% of the original.

## Graphical abstract

Using a collaborative dataset from three populations of Canada lynx in the northwestern United States, we explore the impact of regional variation in animal‐environment relationships on model transferability, as well as how model performance and transferability is affected by the size of the GPS dataset used. Despite the specialist nature of Canada lynx, we found regional differences in lynx‐environment relationships. Model transferability improved as populations became closer in terms of their lynx‐environment relationships. We found substantial redundancy in our large GPS dataset, with predictive performance insensitive to sample sizes above 30% of the original.

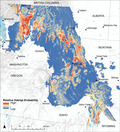

## 1 INTRODUCTION

Species distribution models (SDMs), which compare environmental conditions at presence and background locations and calculate a relative probability of habitat suitability (Elith & Leathwick, 2009), are a useful tool to better understand the distribution of a species' habitat across landscapes (Elith & Leathwick, 2009; Guisan & Thuiller, 2005). These models can provide both an understanding of the specific environmental components that might define a species' habitat as well as generate spatial predictions of distribution at a landscape scale (Elith & Leathwick, 2009). Species distribution models have been used extensively to create maps of predicted habitat (Derville et al., 2018; Gantchoff et al., 2019), evaluate threats from climate change or increased anthropogenic disturbance (Diniz‐Filho et al., 2009; Requena‐Mullor et al., 2019), or consider habitat corridors and connectivity (Zeller et al., 2018). Accurate SDMs are particularly important for landscape‐scale conservation planning given the large‐scale changes associated with climate (Park Williams et al., 2013), anthropogenic alterations (Curtis et al., 2018), habitat loss and fragmentation (Sala et al., 2000), wildfire (Hansen et al., 2010), and insect outbreaks (Kurz et al., 2008). However, one of the limitations faced by SDMs, and indeed all ecological models, is uncertainty about their transferability when applied to novel conditions (Lonergan, 2014; Yates et al., 2018).

When SDMs are implemented across a species' range, they assume a uniform response to the variety of environmental conditions encountered. However, SDMs often encompass multiple, geographically distinct populations which may vary in their responses to local conditions (Barbosa et al., 2009; Habibzadeh et al., 2019; Valladares et al., 2014). Differentiation between individual populations may generate poor model performance outside the model training area, producing erroneous conclusions if that model is applied to other areas. The importance that regional variability plays in SDMs has been demonstrated frequently in plants (O'Neill et al., 2008; Valladares et al., 2014), amphibians (Davies et al., 2019), birds (Habibzadeh et al., 2019), and mammals (Barbosa et al., 2009). Regional variation in intraspecific habitat relationships has been attributed to multiple biological processes, including local adaptation through genetic differentiation (Peterson et al., 2019), biotic interactions (Wisz et al., 2013), or functional responses to differences in habitat availability (Vanreusel et al., 2007). By understanding differences in environmental relationships associated with individual populations, we can improve the development of SDMs, generating improved model predictability and transferability (O'Neill et al., 2008; Vanreusel et al., 2007).

Additionally, SDMs may not generalize geographically because of model over‐fitting, whereby model predictive ability is high in areas where data were collected, but low in areas outside those conditions (Wenger & Olden, 2012). Complex models with excessive environmental covariates, for instance, may result in models which are less generalizable to novel areas (Yates et al., 2018). Similarly, models with large amounts of localized data may not generalize to other landscapes because of the specificity of the species‐environment relationships characterized (Boria & Blois, 2018; Wenger & Olden, 2012). While the impact of sample size on SDMs has been extensively considered, the general concern has been with too little data, rather than too much (Hernandez et al., 2006; Stockwell & Peterson, 2002). However, the recent availability of extensive Global Positioning System (GPS) datasets presents a novel challenge to conventional SDMs as there is little consensus regarding how to treat the large volume of animal relocations (Gantchoff et al., 2019; Li et al., 2017; Magg et al., 2016; Maiorano et al., 2015; Rice et al., 2013; Shoemaker et al., 2018) which may create redundant or spatially correlated nonindependent information with respect to species distributions, particularly if few animals are sampled. Yet, GPS data provide high spatial accuracy, reduced sampling bias, and less species misidentification; all these issues plague the opportunistic sampling schemes common in SDM literature (Aubry et al., 2017; Newbold, 2010). The challenge of modeling distributions of species with large GPS datasets has received little attention (but see Boria & Blois, 2018), but given the availability and benefits of extensive GPS data, an evaluation of the trade‐offs between sampling efficiency and SDM performance is needed.

Our study goals are twofold: (a) evaluate SDM generalizability to model the distribution of Canada lynx (*Lynx canadensis*; hereafter lynx), a federally listed specialist forest carnivore in the contiguous United States, and (b) develop a process to assess GPS data efficiency with respect to SDM predictability and transferability. Lynx rely almost entirely on snowshoe hares (*Lepus americanus*) as a food source (Aubry et al., 2000; Squires & Ruggiero, 2007), and thus are closely tied to boreal forests with high horizontal vegetation cover (Holbrook et al., 2017; Squires et al., 2010). Lynx are an excellent species to assess geographic generalizability of SDMs across populations, because we expect habitat specificity and selection for a narrow range of environmental conditions to result in less intraspecific variation and more habitat generalizability compared to generalist species (Bonthoux et al., 2017; Yates et al., 2018). We used data from three geographically distinct populations at the species' southern range periphery in Washington, Montana, and Wyoming, USA. Our conservation aim was to model the distribution of habitat capable of supporting lynx across the northwestern United States, including areas outside known populations. To inform predictions of SDM generalizability among lynx populations, we first evaluated regional variation in lynx‐environment relationships between populations. We hypothesized that, if regional variation was present, models built on individual populations would perform best for the training population but be less transferable outside that population. We suspected a combined model (using all populations) might perform more poorly on any single population but have higher overall performance across the entire region. We assessed model performance using withheld data as well as an independently collected dataset. To evaluate the efficiency of GPS data in SDMs, we compared model performance and transferability across a range of sample sizes to determine optimal sample size for SDMs when using GPS datasets.

## 2 METHODS

### 2.1 Study areas

**FIGURE 1:**
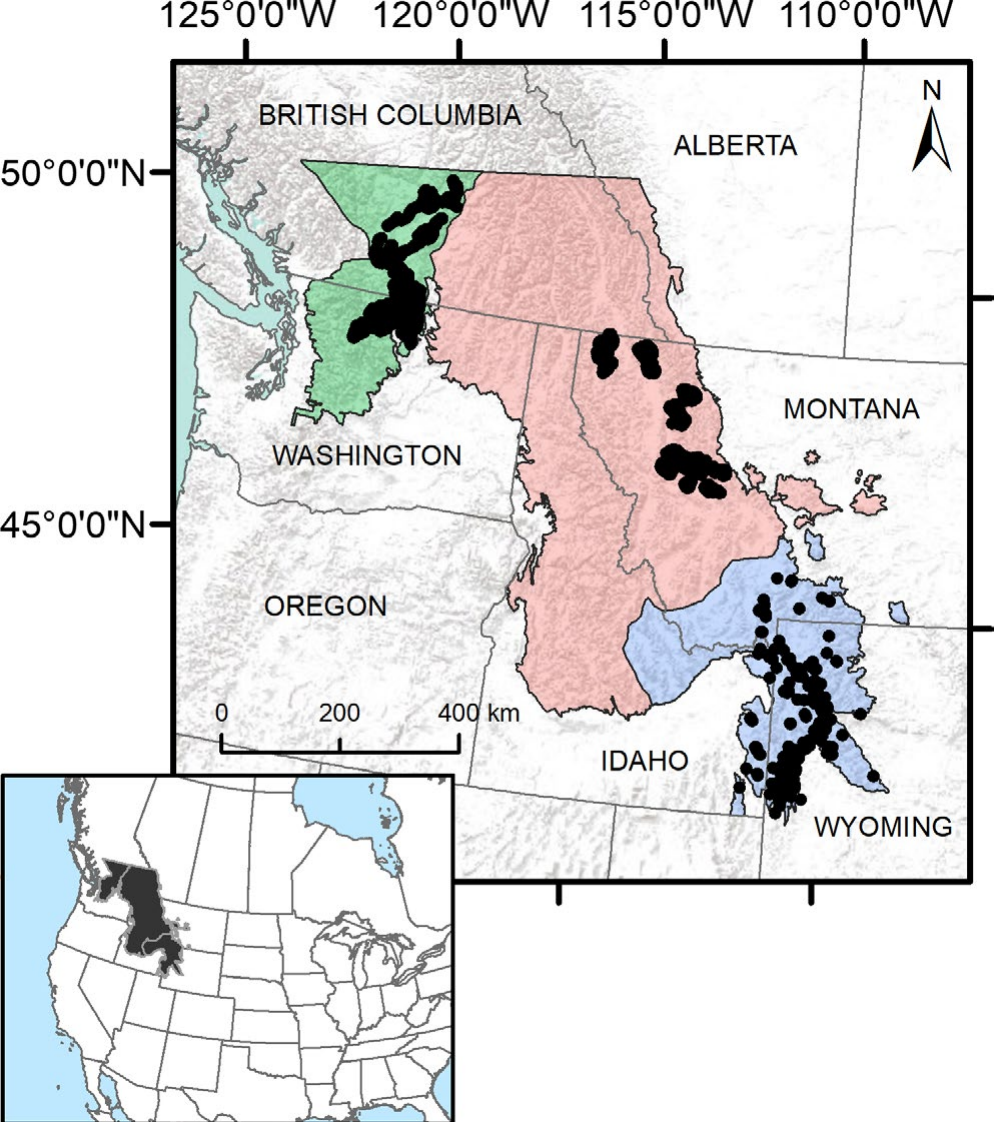
Species distribution modeling extent for Canada lynx covering portions of Washington, Idaho, Montana, and Wyoming, USA, and British Columbia and Alberta, Canada. Black dots indicate lynx GPS locations; color shading indicates the background extent used for each population‐level model (green = Washington, red = Montana, blue = Wyoming). Inset shows location of modeling extent in North America. Background image sources ESRI, USGS, NOAA

Our study area covered a large region in the northwestern United States, including parts of Washington (WA), Idaho (ID), Montana (MT), and Wyoming (WY), as well as the area directly to the north, including parts of British Columbia and Alberta, Canada (Figure 1). We bounded the study area using the level II ecoregion “western cordillera,” which is primarily forested mountains with limited grasslands or other open areas (Omernik & Griffith, 2014). Within our study area were three monitored lynx populations: one in north‐central Washington and into Canada, one in western Montana, and one in northwest Wyoming (Figure 1). These populations are discrete and, though genetic data indicates that north‐south movement renders the contiguous United States and Canada populations panmictic (Schwartz et al., 2002), telemetry data from marked individuals exhibit no east‐west dispersal between populations. Pairwise distances between population centroids were approximately 400 km, 600 km, and 1,000 km for Washington and Montana, Montana and Wyoming, and Wyoming and Washington, respectively. General environmental conditions averaged at lynx locations within each geographic area are given in Table 1; we calculated elevation from a digital elevation model (DEM; U.S. Geological Survey, National Elevation Dataset), and mean annual precipitation, mean annual temperature, and mean snow depth on April 1 from Wang et al. (2016).

**TABLE 1:** Mean and range of environmental conditions averaged across Canada lynx locations at each of the three distinct populations used to make species distribution models across the northwestern United States

	Elevation (m)	Annual precipitation (cm)	Annual temperature (°C)	Snow depth (m)
Washington	1,634 (453–2,452)	76 (60–261)	2.9 (−0.7 to 7.9)	1.3 (0–4.0)
Montana	1,680 (737–2,499)	98 (43–180)	3.4 (0.4–7.0)	1.3 (0–2.8)
Wyoming	2,572 (1,568–3,405)	70 (38–175)	1.3 (−1.1 to 5.9)	1.5 (0–2.8)

### 2.2 Occurrence data

We used GPS data from radio‐collared lynx. Data consisted of 17 individuals (*n* = 21,518 locations) monitored from 2007 to 2013 in Washington, 66 individuals (*n* = 164,612 locations) monitored from 2004 to 2015 in Montana, and 10 individuals monitored from 1996 to 2010 in Wyoming (*n* = 539 GPS locations, *n* = 218 Argos locations). Because of fewer marked lynx in Wyoming, we included both individuals with GPS collars (*n* = 2) and individuals with Argos satellite collars (*n* = 8). We used only Argos locations with spatial accuracy ≤500 m, which was sufficient for our scale of inference. Since the grain of the environmental covariates we used was large (250 m) compared to the resolution of the GPS data, resulting in multiple GPS locations per grid cell, we converted all GPS or Argos locations within a single 250 m cell into a single observation and used this dataset (WA *n* = 7,476, MT *n* = 22,510, WY *n* = 670) as the starting point for all analyses.

### 2.3 Environmental predictors

Environmental predictors were initially selected based on previous knowledge of Canada lynx natural history and ecological relationships (Holbrook et al., 2017; Ivan & Shenk, 2016; Koehler et al., 2008; Maletzke et al., 2008; Squires et al., 2010). We selected 16 climate, topographic, anthropogenic, and vegetative covariates that we expected to be related to Canada lynx distribution (see Appendix A: Table A1 for information on variable selection). To accommodate the temporal period over which our data were collected (1996–2015), we used covariates averaged over the same timeframe whenever possible. Climate variables included mean temperature of the coldest month, winter (December to February) precipitation, summer (Jun to Aug) precipitation, and mean annual relative humidity generated from the ClimateNA v5.10 software package over a period of 1980–2010 with a native resolution of 1 km (AdaptWest Project, 2015; Wang et al., 2016). Heat load (an index of temperature considering aspect and slope), compound topographic index (a steady‐state wetness index), and integrated moisture index (an estimate of soil moisture based on topographic heterogeneity), were created using a 250 m digital elevation model and the Geomorphometric and Gradient Metrics Toolbox (Evans et al., 2014) in ArcMap (Environmental Systems Research Institute, ArcGIS Desktop: Release 10.5.1. Redlands, CA). Snow water equivalent (SWE) and snow depth at 1 km resolution were downloaded from 2003 to 2017 from the National Weather Service's Snow Data Assimilation program (National Operational Hydrologic Remote Sensing Center, 2004) and averaged across years. Minimum snow density was created by dividing snow depth by snow water equivalent (Natural Resources Conservation Service Oregon; United States Department of Agriculture, 2020).

Topographic variables included surface area, an index of topographic ruggedness (Jenness, 2013b), and topographic position index, a measure of the concavity or convexity of a landscape (Jenness, 2013a), created from a 250 m digital elevation model. Vegetation covariates included normalized difference vegetation index (NDVI) from Landsat 5 and 8 imagery averaged during the growing season (1 July to 30 September) from 2000 to 2015, which characterized long‐term vegetation presence and productivity with a 30 m native resolution (Pettorelli et al., 2005). We also calculated standard deviation of percentage of tree cover (Hansen et al., 2013) in a 1 km neighborhood as an index of forest heterogeneity. We considered soil pH, since the wetter conditions of boreal forests would be expected to have lower pH (Hengl et al., 2017), as well as anthropogenic influences of road density (highway, local, and open forest roads) within a 1 km neighborhood (OpenStreetMap Foundation, 2017) and night light intensity, an index of anthropogenic presence compiled from nighttime lights visible from cities and towns from 1996 to 2011 (National Oceanic & Atmospheric Administration, 2014). We resampled all predictors to a 250 m resolution and reprojected to the Albers Equal Area projection. Pairwise correlations between predictors are given in Appendix B; all covariates were correlated *r* ≤ |0.7|.

### 2.4 GPS data efficiency

To explore the impact of sample size on model performance and determine the optimum sample size of GPS locations for model calibration, we performed a sensitivity analysis of predictive performance across varying sample sizes. From the original dataset (WA *n* = 7,476, MT *n* = 22,510, WY *n* = 670), we randomly sampled a percentage of each population (MT, WA, or WY) from 5% to 100% of the original sample size in increments of 5%. For each sample size, we selected an equal number of background locations within the extent of each population and fit the same ensemble model including 11 topographic and climate variables and six modeling algorithms, and evaluated models using withheld and independent datasets (see below for full modeling and validation details). We compared model performance using AUC (Marmion et al., 2009) to assess model predictive ability as well as transferability across sample sizes. We used the outcome from the sample size simulation to determine optimum trade‐off between model performance and data parsimony, with the assumption that the sample size reached before a drop in performance had little to no data redundancy or spatial correlation, and adopted this sample size (WA *n* = 2,243, MT *n* = 6,753, WY *n* = 540) for each GPS dataset for the remainder of our analyses.

### 2.5 Regional variation between populations

To explore the hypothesis that regional variation was present between populations, we performed a principal component analysis (PCA; Hällfors et al., 2016). If regional variation was present, we expected to observe distinct clustering of the three populations within the PCA dimensions. We used all 16 covariates from our models and ran the PCA on the lynx locations from the dataset used in the SDM modeling process using the “PCA” function from the R package “FactoMineR” (Lê et al., 2008). We plotted lynx locations with 95% confidence intervals of clustering on the first two dimensions of the PCA to visualize grouping of the populations. We used the correlation between individual covariates and the first two principal components to inform which covariates were contributing most to each component. This allowed us to identify the environmental gestalt associated with each population. We hypothesized that populations similar in principal component space would be more transferable to each other than populations farther away, regardless of geographic distance, because of environmental similarity.

### 2.6 SDM modeling approach

#### 2.6.1 SDM development

**FIGURE 2:**
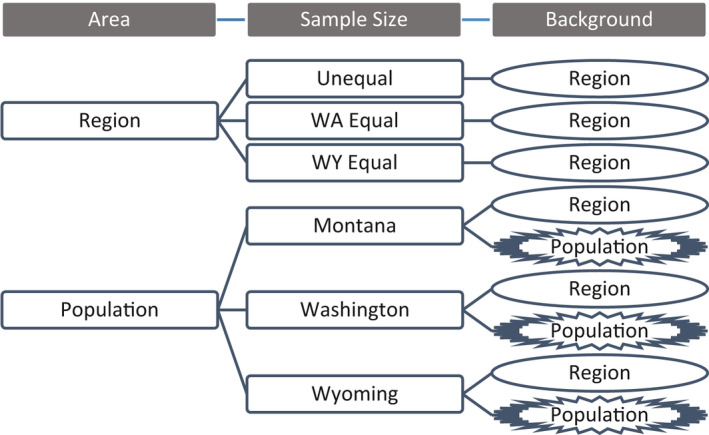
Schematic showing the number of species distribution modeling scenarios performed for the study; models were performed on either populations or the entire region, with varying sample sizes, and different extents for the selection of background locations

We constructed separate SDMs for each individual population and a regional model with all combined populations. Since one of our modeling goals was to explore the effects of data efficiency given the use of large GPS datasets, we considered three sample size scenarios for models from the entire region: unequal sample sizes from each region (“Unequal,” based on initial size of each population dataset; WA *n* = 2,243, MT *n* = 6,753, WY *n* = 540), equal sample size where possible based on Washington (“WA Equal,” MT and WA *n* = 2,243, WY *n* = 540), and equal sample size based on Wyoming (“WY Equal,” all sample sizes reduced to equal WY sample size *n* = 540; Figure 2). Presence locations for reduced datasets were chosen randomly from the initial population dataset. Since SDMs are often sensitive to the extent and locations chosen as randomly distributed background data (Iturbide et al., 2018), we also considered two scenarios to explore the effect of background extent of individual population models on model prediction and transferability: background data from either the entire region or an area associated with only the local population (Figures 1 and 2). We split our combined regional study area into three population areas subjectively based on landscape features such as large rivers and nonforested spaces that we hypothesized would be difficult for lynx to cross (Figure 1). This resulted in a total of 9 modeling scenarios (Figure 2).

Background locations were initially sampled at approximately 1 point per 1.5 km^2^ across the study area to ensure adequate coverage. We then subsampled from these points to create a background sample equal to the number of lynx GPS locations per population, depending on which scenario was being modeled. We used the “biomod2” package (Thuiller et al., 2009) in program R v. 3.6.0 (R Core Team, 2019) for all distribution modeling, and six modeling algorithms were selected to include a range of regression (Boosted Regression Trees, Multiple Adaptive Regression Splines, Generalized Linear Models, and Generalized Additive Models) and machine‐learning methods (Random Forest, Maxent) commonly used in an SDM context. To decrease variability resulting from a random sampling of background locations, we ran each model 10 times with a different random sample of background replicates each time (Barbet‐Massin et al., 2012). This resulted in 60 models per scenario, which were combined into a weighted average based on area under the curve (AUC) of the receiver operating characteristic (ROC), so that better‐performing models contributed more to the final ensemble, with the threshold for inclusion greater or equal to the median AUC calculated from all 60 models. Ensemble modeling has demonstrated equal or superior predictive performance relative to single models (Hao et al., 2020; Marmion et al., 2009).

#### 2.6.2 SDM validation

We assessed model predictive performance using AUC (Fielding & Bell, 1997), the continuous Boyce index (Hirzel et al., 2006), and the minimal predicted area (MPA; Engler et al., 2004). The AUC considers model discriminatory ability at all possible thresholds; we used the partial‐area ROC (Peterson et al., 2008), which uses the proportion of background area predicted as present, rather than absence locations, as the x‐axis metric. This variation makes the AUC metric more applicable to SDMs, since the models are based on presence and background (rather than presence and absence) data. For background data, we again randomly sampled the entire study area at a density of 1 point per 10 km^2^ to provide a spatially well‐distributed sample. The continuous Boyce index quantifies the delineation of capable habitat using a Spearman rank correlation between the ratio of predicted to expected number of presence locations and mean habitat capability grouped into equal‐area bins (Boyce et al., 2002; Hirzel et al., 2006). MPA uses a chosen threshold (in our case 90% of presence locations) applied to the prediction surface to determine extent of the area above this threshold; this evaluation provides a metric of model efficiency, illustrating the trade‐off between correctly identifying presence locations while doing so with a minimum of predicted area. We used the R package “pROC” (Robin et al., 2011) to calculate AUC and “ecospat” (Di Cola et al., 2017) to calculate the Boyce index.

We used two datasets for model validation: a withheld dataset consisting of GPS data that were not used in model calibration (WA *n* = 5,233, MT *n* = 15,757, WY *n* = 130) and an independent dataset compiled from diverse data sources (WA *n* = 52, MT *n* = 445, WY *n* = 23, ID *n* = 103, Canada *n* = 27), including noninvasive genetic sampling (*n* = 375), camera traps (*n* = 71), den locations (*n* = 80), incidental sightings and mortalities (*n* = 31), other Argos locations (*n* = 62), and two GPS collared individuals that were outside of the three main populations of interest and thus included only as validation data (*n* = 27). We assessed model performance for each SDM within the population on which it was calibrated, the geographically separate populations to determine model transferability, and the entire region (all three populations combined). Additionally, for only the best‐performing (most predictive) model, we also assessed relative contribution of each environmental covariate to better understand what factors were contributing to modeled lynx distribution (Hällfors et al., 2016). We evaluated the importance of covariates by permuting a single variable, generating model predictions, and calculating the correlation between these permuted predictions and the original model predictions; if a variable was important, model predictions would be altered, and correlation between predictions would be low when the variable was permuted (Thuiller et al., 2009). Since we used an ensemble of six modeling techniques, each variable was given six measures of importance, which we combined in a single boxplot for illustrative purposes.

#### 2.6.3 SDM mapping

To identify key conservation areas for sensitive species, like lynx, that occupy extensive ranges, we generated predictions from the top‐performing SDM in both continuous and categorical formats. Continuous predictions provide a detailed look at the relative habitat suitability of lynx across the study area, while a categorical map provides simplified predictions that may be more useful to managers responsible for conservation planning (Freeman & Moisen, 2008). For example, an important application for the lynx SDM developed here is to generate habitat predictions in areas between the three main populations. Therefore, we applied a threshold to our top‐performing model chosen to include 90% of lynx GPS locations (composed of reproductive populations on home ranges) as “high” probability lynx habitat and a threshold chosen to include 85% of independent data as “medium” probability lynx habitat. The independent location data for lynx included incidental sightings of animals outside the range of core populations and therefore may represent a larger array of behaviors and thus of habitat use. We chose the 90% and 85% cutoff for high and medium lynx data, respectively, to maintain a high conservation standard with low acceptable error (here 15% or less) and using values consistent with data cut‐offs for home‐range delineation and various habitat thresholds in the literature (Börger et al., 2006; Freeman & Moisen, 2008). However, to acknowledge a range of potential thresholds for different conservation goals, we also considered two thresholds that bracketed these criteria, one of 95% lynx locations and 90% independent data, and a second of 85% lynx locations and 80% independent data.

## 3 RESULTS

### 3.1 GPS data efficiency

**FIGURE 3:**
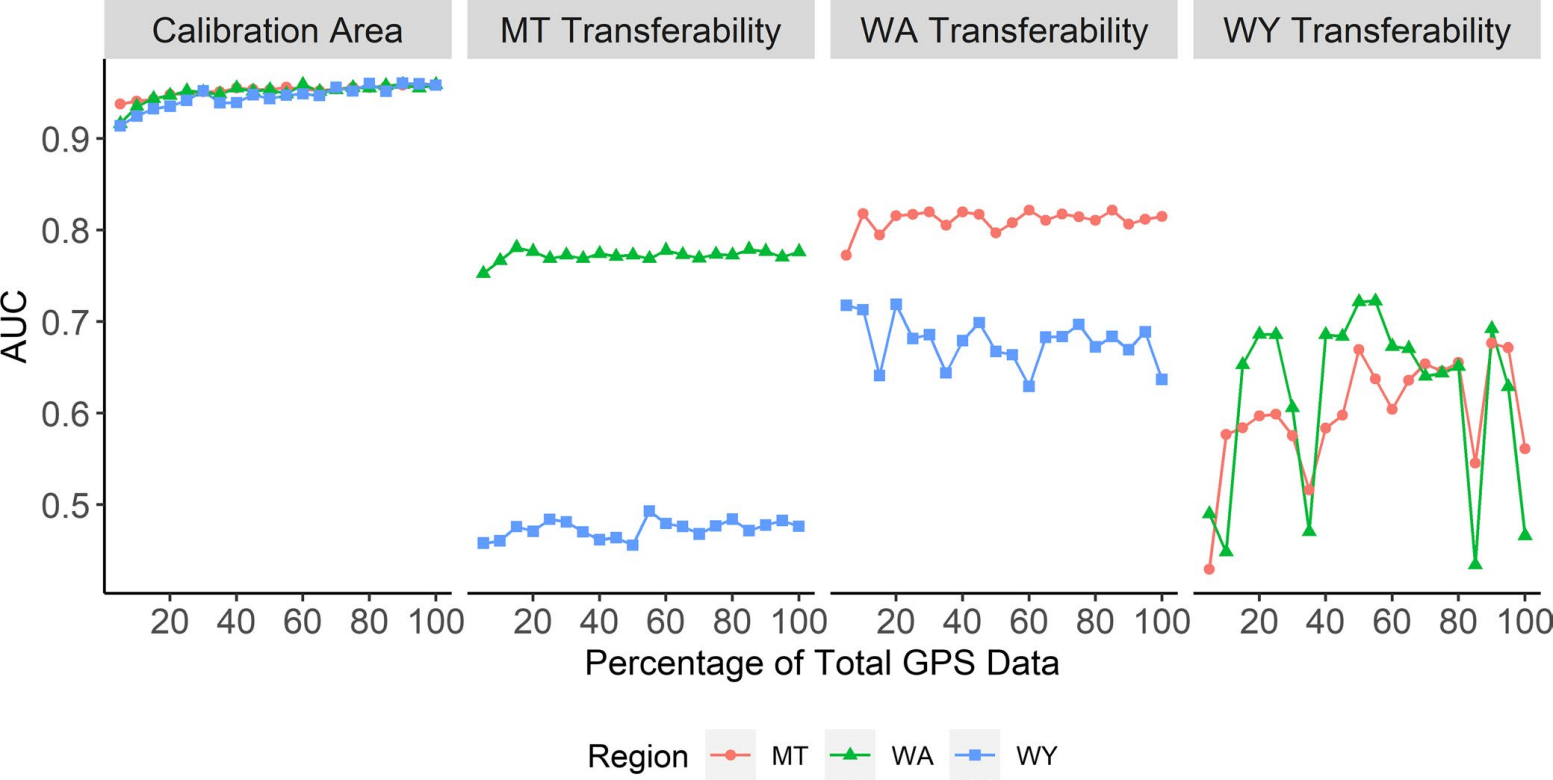
Performance of species distribution models, as measured by the area under the curve (AUC), for a range of sample sizes from 5% to 100% of the original Canada lynx GPS dataset. The first panel shows model performance when evaluated on data within the area that the model was trained on (Calibration Area). The second through fourth panels show the performance of models trained on a given population (“MT” = Montana, “WA” = Washington, “WY” = Wyoming) when transferred to the remaining populations. For example, “WA Transferability” shows models calibrated in Washington but tested on data from Montana and Wyoming

**FIGURE 4:**
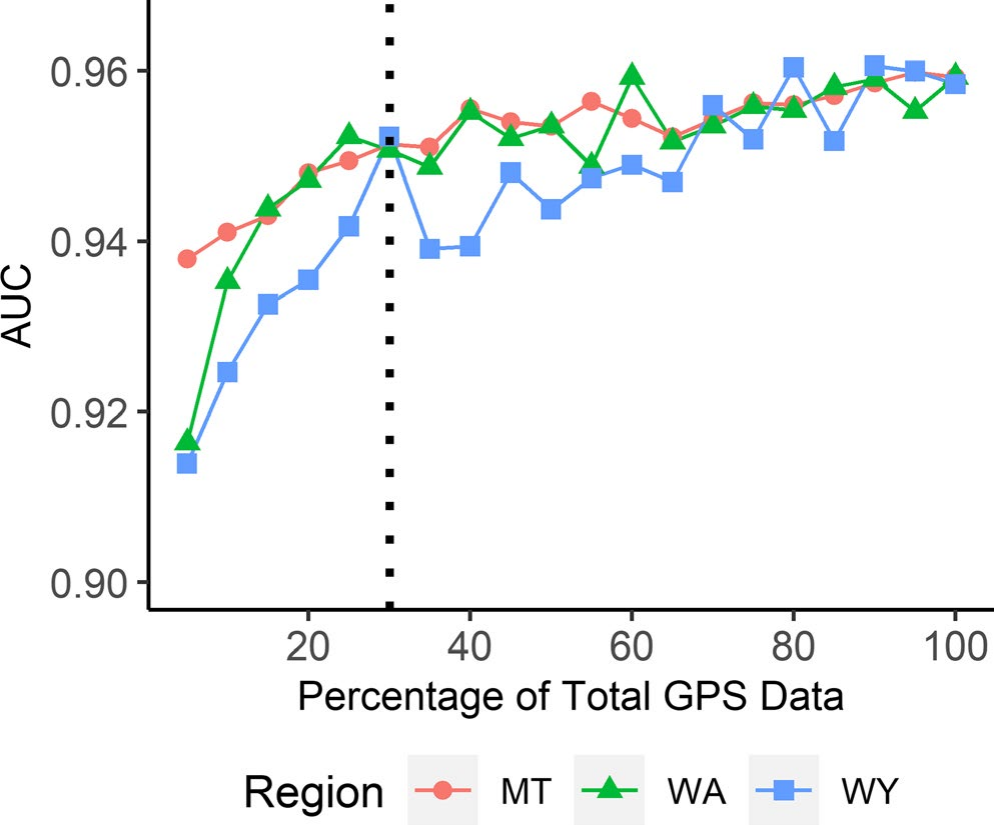
Performance of species distribution models, as measured by area under the curve (AUC), for a range of sample sizes from 5% to 100% of the original Canada lynx GPS dataset. This figure shows a close‐up of the first panel from Figure 3, of model performance when evaluated on data within the area that the model was calibrated on. Model performance for each region (“MT” = Montana, “WA” = Washington, “WY” = Wyoming) improves steeply from 5% to approximately 30%, but plateaus thereafter

We found model performance to be mostly insensitive to sample size. Models trained on 100% of GPS location data were less than 0.05 AUC (<5% gain) better than those trained on only 5% when tested on withheld data (MT: 5% AUC = 0.938, 100% AUC = 0.959; WA: 5% AUC = 0.916, 100% AUC = 0.959; WY: 5% AUC = 0.914, 100% AUC = 0.958; Figure 3). Independent data validation showed even less difference, with a gain of 0.03 AUC (<4% gain) or less (MT: 5% AUC = 0.840, 100% AUC = 0.855; WA: 5% AUC = 0.822, 100% AUC = 0.858; WY: 5% AUC = 0.800, 100% AUC = 0.803). Despite large differences in sample size between populations, we did not find pronounced differences in AUC between populations at similar sample size percentages (Figure 4). For instance, at 5% of the data, Wyoming's model contained *n* = 34 locations and had an AUC of 0.800 with independent data, while Washington had *n* = 374 and an AUC of 0.822, and Montana had *n* = 1,126 and AUC of 0.840. Taken together, these results indicate that model performance within the calibration area was robust to small sample size and relatively unaffected by up to 33‐fold differences in absolute number of presences. Additionally, while model performance plateaued above ~30% data, we did not detect any drop in model performance up to the maximum sample size of *n* = 22,510 in Montana. While differences in AUC between sample sizes were small, the biggest gain in AUC appeared between 5% and 30% before reaching a plateau (Figure 4). Thus, for further modeling, we considered a sample size of 30% of the data (WA: *n* = 2,243, MT: *n* = 6,753), to be the appropriate balance between model performance and data redundancy. However, we found the Wyoming population increased in model performance until approximately 80% of the dataset was included. We assumed this was a function of the limited data that defined lynx in Wyoming compared to other populations, so we used 80% of the Wyoming data (WY: *n* = 540) in subsequent analyses to maximize model predictive performance for the Wyoming population (Figure 4).

The percent of data used had little effect on model transferability across populations (Figure 3), but with some differences between individual populations. The model created with data from only the Washington population had the highest predictive performance in the other two populations, with a mean AUC of 0.811 on withheld data in Montana (5% = 0.772, 100% = 0.815) and 0.678 in Wyoming (5% = 0.718, 100% = 0.637). The models built from the Montana population were less transferable but more stable in performance across the gradient of sample size, most likely due to the large absolute sample size of Montana. Montana models performed well in Washington (mean AUC = 0.772, 5% AUC = 0.753, 100% AUC = 0.777) but poorly in Wyoming (mean AUC = 0.473, 5% AUC = 0.458, 100% AUC = 0.476; Figure 3). Models built in Wyoming were inconsistent in transferability across sample sizes (Figure 3); transferability of Wyoming models was similarly poor in Montana (mean AUC = 0.601, 5% AUC = 0.430, 100% AUC = 0.561) and Washington (mean AUC = 0.618, 5% AUC = 0.490, 100% AUC = 0.466).

### 3.2 Regional variation between populations

**FIGURE 5:**
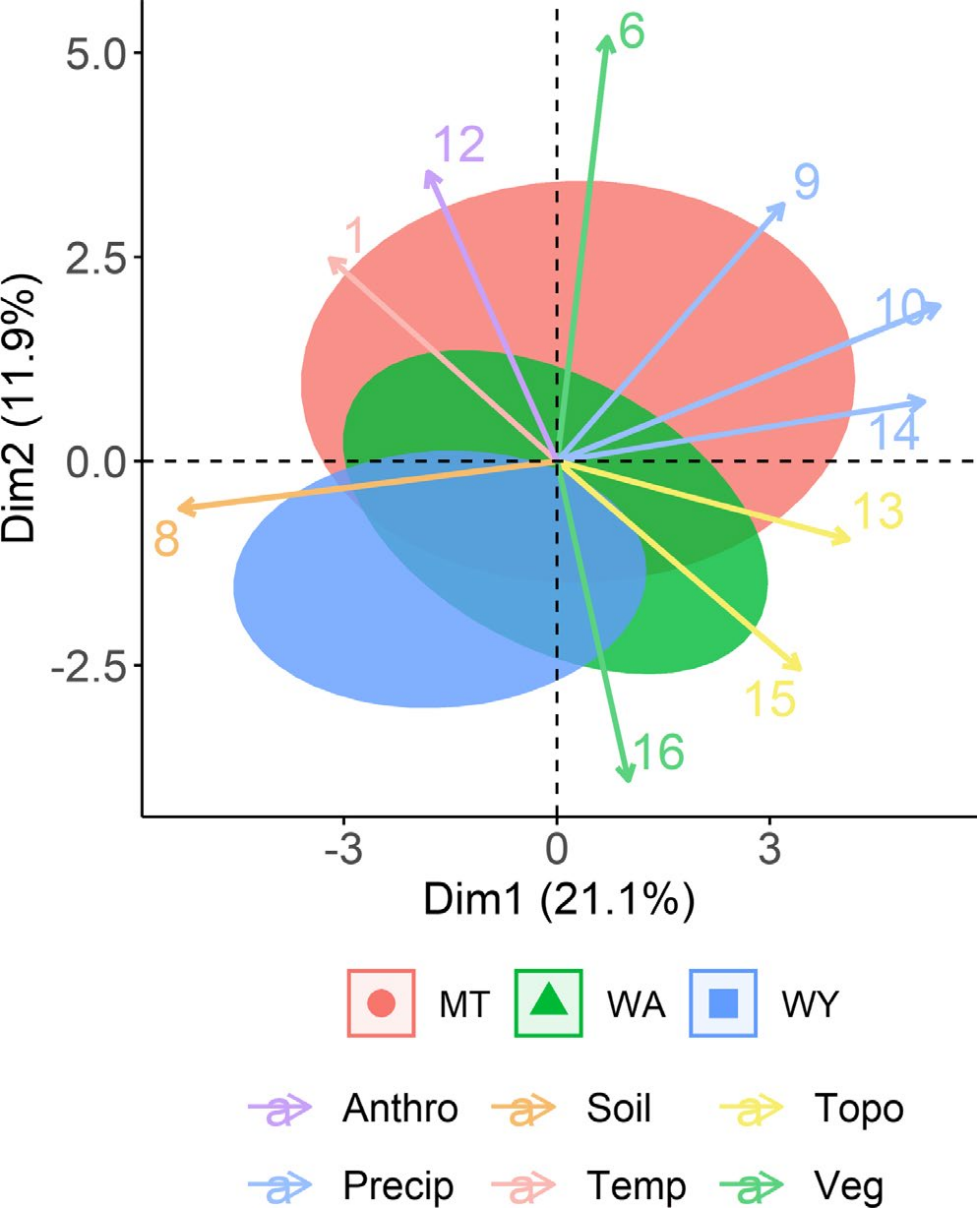
The results of a principal components analysis across the three Canada lynx populations using the 16 climate, topographic, vegetation, and anthropogenic covariates included in species distribution models. The red ellipse represents the 95% confidence interval around the Montana population, green Washington, and blue Wyoming. Arrows represent correlation between each covariate to the principal component axes; arrows are colored by type of covariate (Anthropogenic, Soil, Topography, Precipitation, Temperature, Vegetation), and only the top 10 contributing covariates are shown. The direction of the arrow indicates to which dimension the covariate contributes most. Covariate arrows are labeled by number for readability: 1 = Compound Topographic Index, 6 = NDVI, 8 = Soil pH, 9 = Summer Precipitation, 10 = Winter Precipitation, 12 = Road Density, 13 = Surface Area, 14 = Snow Water Equivalent, 15 = Topographic Position Index, 16 = Forest Heterogeneity. Percentage by axes show how much variation is explained by the first (Dim1) and second (Dim2) dimension in the principal components

Counter to our expectations for a specialist species, some regional variation was present across the three populations of lynx as demonstrated through clustering in PCA space. Wyoming and Montana populations were the most differentiated, while Washington exhibited a combination of characteristics between Wyoming and Montana (Figure 5). The PCA explained 33% of the variation in the first two axes, with PC1 dominated by precipitation‐related covariates (summer and winter precipitation, relative humidity, and soil pH) and PC2 dominated by vegetation‐related covariates (long‐term NDVI, forest heterogeneity, and road density; Appendix C: Tables C1 and C2). The Wyoming population was grouped on the PCA axes based on less moisture, lower long‐term NDVI, and more forest heterogeneity than the Montana population. Interestingly, Washington fell in between Montana and Wyoming along these axes, despite its relative isolation in geographic space (Figure 1).

### 3.3 Lynx SDM performance

**FIGURE 6:**
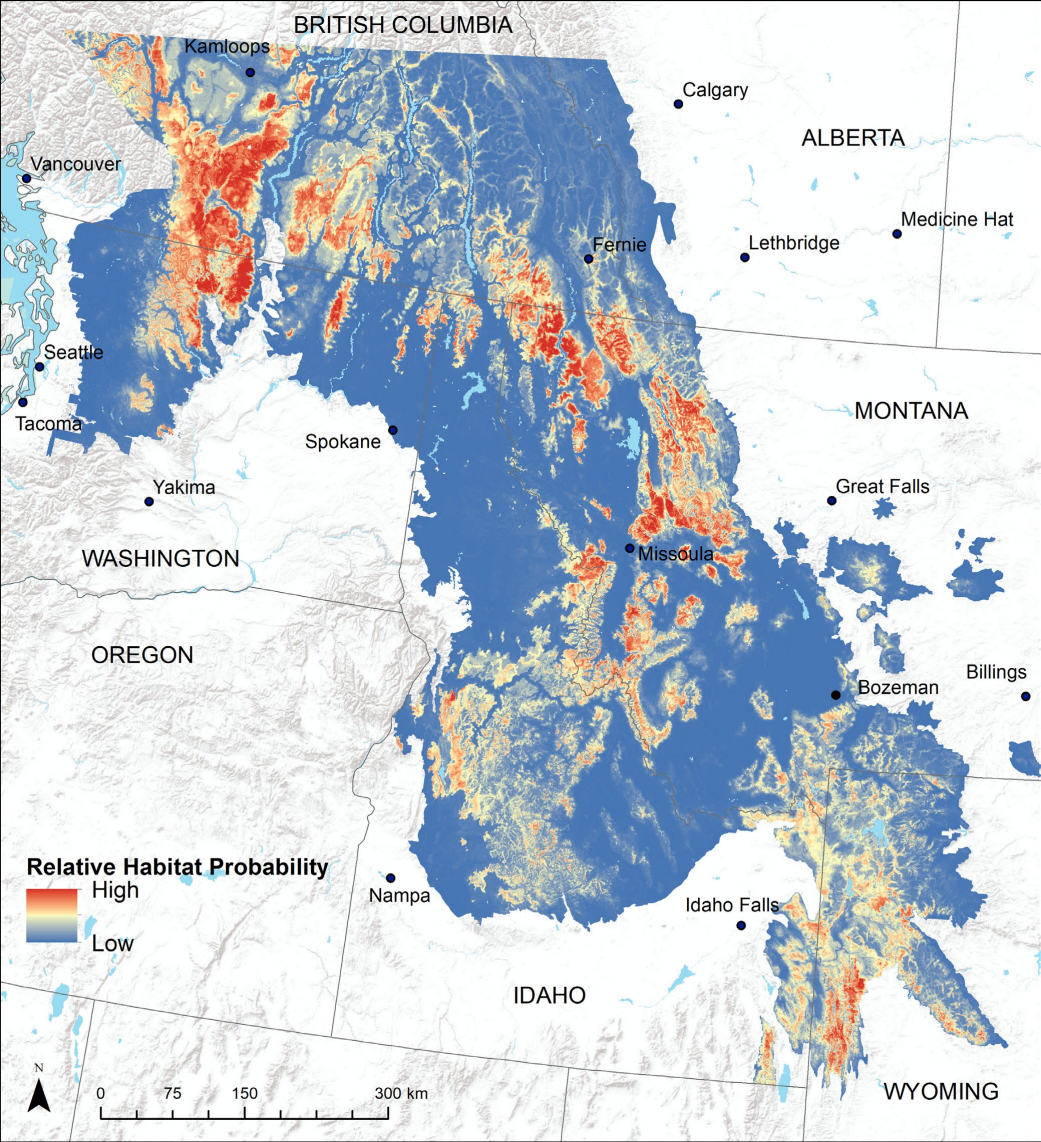
Spatial predictions of Canada lynx relative habitat probability across the study region in the northwest United States, as predicted by the top‐performing species distribution model. Background image sources ESRI, USGS, NOAA

**FIGURE 7:**
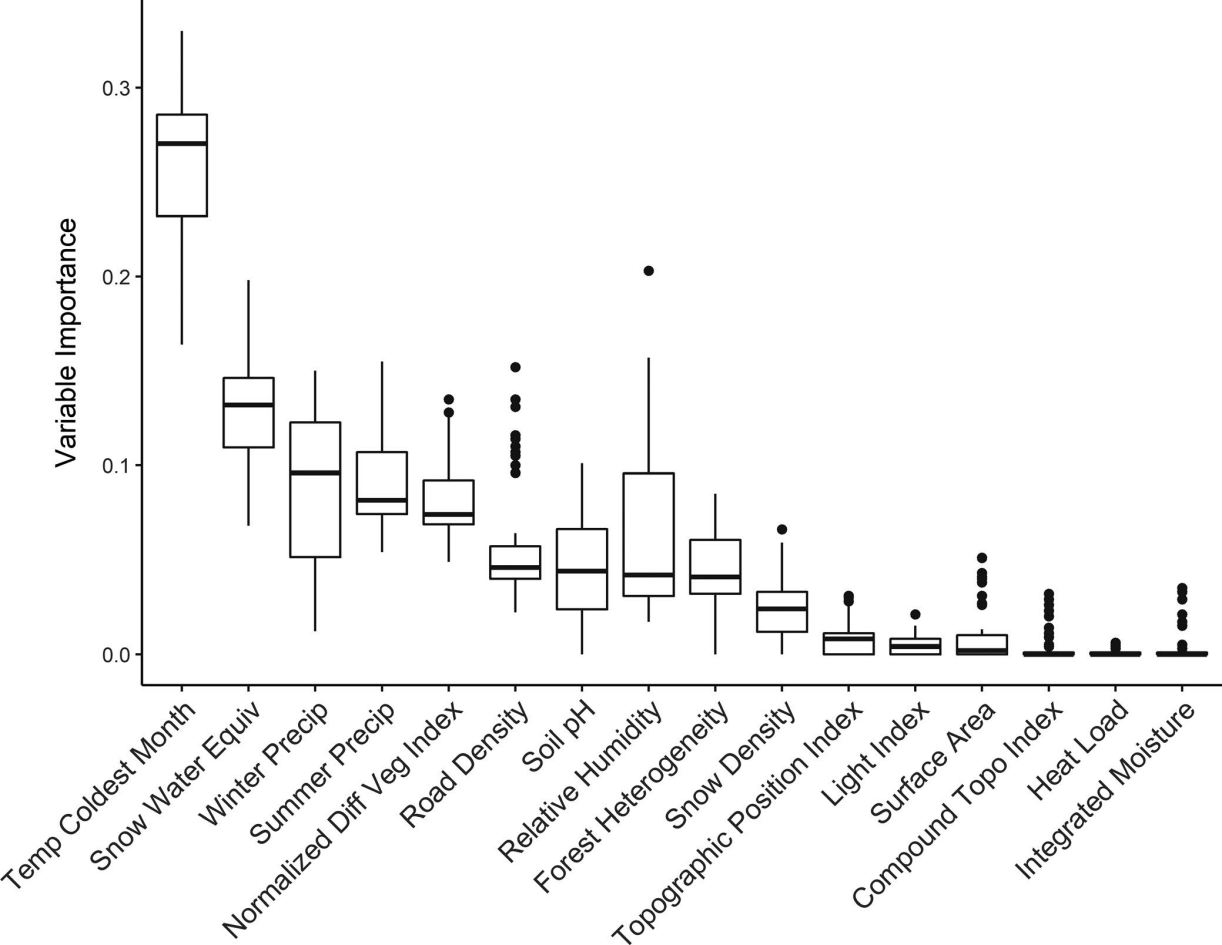
Estimated variable importance of each covariate to the best‐performing species distribution model. Variable importance was estimated by permuting each covariate in turn, generating predictions, and comparing predictions to those from the original, unpermuted model. If a covariate was important, predictions would be changed and the correlation between sets of predictions would be lower

Consistent with the PCA results, individual lynx population models performed well in the area from which they were developed and were less transferable to other populations (Table 2). Based on model performance assessed on both withheld and independent data, the regional model that used 30% of Washington data and a Montana sample size to match (“WA Equal,” Table 2) was the most predictive of lynx use locations across each population and the entire region combined (see Appendix D for validation results for continuous Boyce Index and MPA). Individual population models made from 30% of the data from each population were slightly more predictive for Montana (AUC = 0.981) and Washington (AUC = 0.959) than the regional model (MT AUC = 0.974, WA AUC = 0.954), but the “WA Equal” regional model performed best in the Wyoming population and across all three populations together (Table 2). Regional models from the three combined populations were consistent in performance when tested separately on each population and exhibited good predictive performance of withheld data (AUC > 0.90) in each population and good predictive performance of independent data (AUC > 0.80) in each population (Table 2). Spatial predictions from the “WA Equal” model matched well with our expectations of lynx habitat and demonstrated areas of high habitat probability in the areas with known reproductive lynx populations as well as smaller islands of probable habitat in areas between populations (Figure 6). Covariates of greatest relative importance were primarily related to snow and precipitation, with mean temperature in the coldest month contributing the most to model predictions, and lesser contributions from snow water equivalent, precipitation in summer and winter, and long‐term NDVI (Figure 7). For population‐specific models, background extent (population versus region) had very little effect on model performance within the calibration area, but model transferability was better for models made with population‐level backgrounds (Table 2).

**TABLE 2:** Model validation, as measured with AUC, for all species distribution models generated for Canada lynx in the northwestern United States

Validation data source	Data location	Model being tested	Background	Performance in			
				MT	WA	WY	Region
Withheld	Region	Unequal	Region	0.977^b^	0.937	0.927	0.939
		WA equal	Region	0.974	0.954^b^	0.973^a^	0.969^a^
		WY equal	Region	0.951	0.929	0.945	0.950^b^
	Population	MT	Region	0.970	0.790	0.540	0.722
		MT	Population	0.981^a^	0.792	0.580	0.781
		WA	Region	0.701	0.946	0.664	0.684
		WA	Population	0.786	0.959^a^	0.781	0.862
		WY	Region	0.535	0.785	0.952	0.692
		WY	Population	0.641	0.469	0.960^b^	0.764

				MT	WA	WY	Region
Independent	Region	Unequal	Region	0.833	0.880	0.912^b^	0.865
		WA equal	Region	0.834	0.884^b^	0.922^a^	0.883^a^
		WY equal	Region	0.821	0.854	0.910	0.868^b^
	Population	MT	Region	0.857^a^	0.766	0.832	0.768
		MT	Population	0.851^b^	0.771	0.824	0.799
		WA	Region	0.652	0.889^a^	0.693	0.683
		WA	Population	0.699	0.863	0.868	0.788
		WY	Region	0.524	0.710	0.791	0.624
		WY	Population	0.624	0.610	0.819	0.734

Note

Values in each column marked with a superscript “a” indicate best model performance in that population, superscript “b” indicate second best.

### 3.4 SDM mapping

**FIGURE 8:**
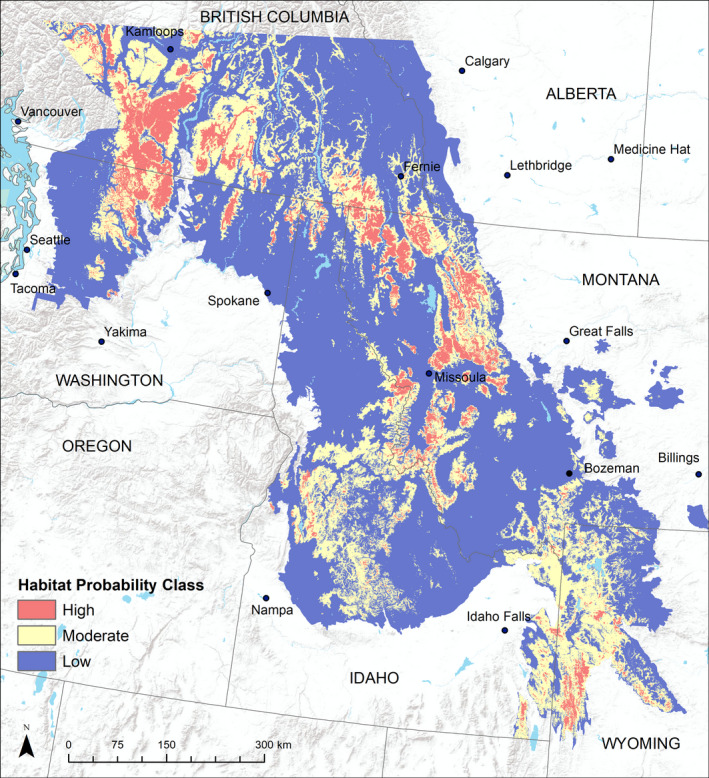
Categorical spatial predictions of Canada lynx relative habitat probability across the study region in the northwest United States, as generated by the top‐performing species distribution model. Model thresholds are based on correctly assigning 90% of Canada lynx withheld GPS locations for the “High” category and 85% of independent lynx locations for the “Moderate” category. Background image sources ESRI, USGS, NOAA

**FIGURE E1:**
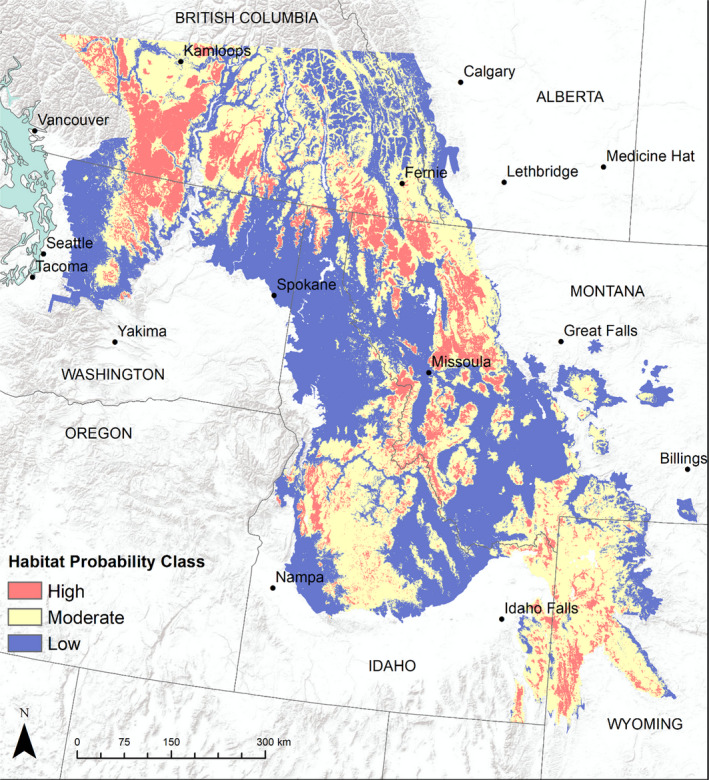
Categorical spatial predictions of Canada lynx relative habitat capability across the study region in the northwest United States, as generated by the top‐performing species distribution model. Model thresholds are based on correctly assigning 95% of Canada lynx withheld GPS locations for the “High” category and 90% of independent lynx locations for the “Moderate” category. These thresholds provide a more liberal delineation of lynx habitat than the 90%/85% thresholds provided in the main paper

**FIGURE E2:**
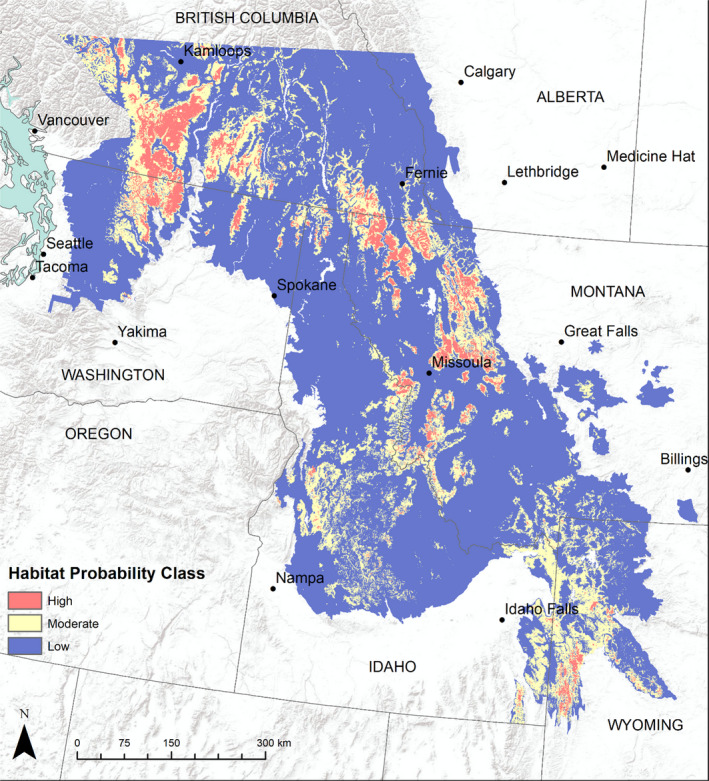
Categorical spatial predictions of Canada lynx relative habitat capability across the study region in the northwest United States, as generated by the top‐performing species distribution model. Model thresholds are based on correctly assigning 85% of Canada lynx withheld GPS locations for the “High” category and 80% of independent lynx locations for the “Moderate” category. These thresholds provide a more conservative delineation of lynx habitat than the 90%/85% thresholds provided in the main paper

Our best‐performing SDM generated predictions consistent with known lynx habitat use (Mckelvey, 2000), with Canada lynx patchily distributed in mountainous areas throughout the Pacific Northwest and the Greater Yellowstone Area (see Figure 8 for details). Categorical predictions created by 90% and 85% threshold values when applied to the “WA Equal” model delineated the location of habitat most likely to be selected by lynx in a reproductive population (“high” probability habitat) and habitat that was less favorable but potentially still used by lynx (“moderate” probability habitat), particularly for connectivity or as part of a matrix with “high” and “low” probability habitat (Figure 8). We delineated 34,930 km^2^ of “high” probability habitat and 125,580 km^2^ of “moderate” probability habitat across the study area. By state, Montana had the largest area of “high” habitat, with 11,961 km^2^, followed by Washington (4,411 km^2^), Idaho (2,497 km^2^), and Wyoming (2,424 km^2^). Differences in amount of area in each category were more pronounced with changes in the threshold generated from independent data, since this dataset included more variation in habitat use (Appendix E: Figures E1 and E2).

## 4 DISCUSSION

Accurate representations of species distributions are increasingly important given the many challenges facing wildlife today. Habitat loss or fragmentation (Hornseth et al., 2014), a changing climate (Zielinski et al., 2017), and negative wildlife‐human interactions (Reilly et al., 2017) all serve to increase the need for conservation of important habitat. Yet the delineation of important habitat is still sometimes unknown, causing conservation actions to be misdirected and wasting the limited resources available. Here, we used data from multiple Canada lynx populations across the northwestern United States and southern Canada, considered niche differentiation and model transferability, and created a highly predictive model of lynx habitat, validated using withheld and independent data. This model provides a refined depiction of lynx habitat that will facilitate the application of conservation management to areas most relevant to Canada lynx.

We expected generalizability between individual lynx population models given the known habitat specificity of lynx but found that, while lynx exhibit narrow habitat selection (Holbrook et al., 2017; Squires et al., 2010), there was enough variation in local animal‐environment relationships to limit transferability of any single population model to our entire inference area. Regional models built using data from all populations combined, however, performed strongly across the entire study area, generated predictions for areas that were outside the three main populations and thus lacked data, and performed comparably to individual population models. Our use of principal components analysis (PCA) to examine regional variation between populations revealed differences and similarities between populations, and thus provided informed predictions of model transferability. The use of GPS data in our work resulted in models with very high predictive accuracy, which was maintained above 0.90 AUC even when data were reduced to approximately 5% of their original sample size.

SDMs are often constructed with opportunistic data collected across large spatial extents or with intensive data collection across smaller extents (Aubry et al., 2017; Thuiller et al., 2006). Few studies have the resources required for extensive data collection at multiple locations across a large area (Bonthoux et al., 2017). However, we combined GPS data from multiple collaborators to directly assess regional differences in habitat selection across populations within a large spatial area. We believe that large‐scale species distribution modeling will increasingly benefit from similar collaborative approaches for creating accurate, regional‐scale suitability models for other species and regions, given the widespread prevalence of GPS monitoring of a range of species by academic, government, and nonprofit institutions.

We found that individual population models performed well for a given population but were less predictive when generalized across the region, consistent with the presence of regional variation in animal‐environment relationships. This result is in line with other studies testing variation in habitat selection across regions or populations. For instance, Torres et al. (2015) demonstrated strong predictive performance of SDMs within individual islands of gray petrels (*Procellaria cinerea*) but weak performance across islands, while McAlpine et al. (2008) found that multiscale models of koala (*Phascolarctos cinereus*) habitat performed more poorly cross‐regionally than within the region of model training. A potential explanation for this is differences in small‐scale habitat availability (Habibzadeh et al., 2019; McAlpine et al., 2008; Torres et al., 2015) that manifest as slightly different realized niches between populations (Soberón & Nakamura, 2009; Torres et al., 2015). Our PCA results demonstrated differences in the environmental conditions used by lynx in each of the three populations, with the degree of difference reflected in their transferability to one another. For instance, the Washington population was located between Montana and Wyoming in PCA space, and this overlap in environmental similarity was reflected in the greater transferability of this model to the Wyoming and Montana populations.

Generalizability of SDMs is also predicted to be related to specificity in diet or habitat selection (Bonthoux et al., 2017; Yates et al., 2018), although this pattern appears to be born out in some species and not others. A similar lack of transferability in habitat selection was observed in koalas (McAlpine et al., 2008), a specialist on eucalyptus leaves, while the opposite pattern was found in several species of European birds living in mixed agricultural land, which demonstrated increased model transferability with habitat specialization (Bonthoux et al., 2017). Specialists are generally predicted to select a narrower range of environmental conditions (Kassen, 2002; Peers et al., 2012), and thus are predicted to favor homogenous environments with resource use similar and transferable across populations. Canada lynx reliance on snowshoe hares as prey make them similarly reliant on the environmental conditions that favor hares (Ivan & Shenk, 2016; Squires et al., 2010). Previous works show that lynx select boreal forest environments with deep snow and high horizontal cover (Holbrook et al., 2017; Mowat et al., 2000; Squires et al., 2010), leading to predicted transferability of SDMs. Instead, models from each individual population had marginal fit when applied to geographic areas outside their training location. One possible explanation is that lynx may use alternate prey when necessary; while their dependence on hares is well known, when hare abundance is low they may turn to alternative prey such as blue grouse (*Dendragapus obscurus*) or red squirrels (*Tamiasciurus hudsonicus*) (Ivan & Shenk, 2016), and thus differ somewhat in habitat use. Alternatively, while the populations sampled may vary in some environmental characteristics, they may be similar enough in features important to hares, such as high horizontal cover in mature forests (Squires et al., 2010), that lynx can find adequate food while still exhibiting habitat differentiation. The lynx population in Wyoming, for instance, is located in habitat that appears strikingly similar in forest structure and horizontal cover to lynx habitat in Montana (J. Squires, pers. com.). Additionally, the lynx in Wyoming that were monitored with Argos collars were partly comprised of individuals originally reintroduced from Canada to Colorado and had exhibited long‐distance post‐reintroduction movements (Devineau et al., 2010). These animals might therefore have been exhibiting atypical habitat selection, which may have included a less specialized pattern of selection, possibly also contributing to the low transferability of the Wyoming model.

Interestingly, despite differences in animal‐environment relationships between populations, the regional model which included data from all populations performed well across the entire study area. Given the lack of generalizability demonstrated by the individual population models, we might expect that a SDM created from all populations would perform more poorly in any given population than a model created only on those data (Torres et al., 2015). Instead, the regional model performed better than the individual population model for Wyoming and was nearly indistinguishable in performance from population‐level models for Washington and Montana. The strong performance of the regional model might be explained by the larger geographic range that it sampled. Sampling a larger portion of the range is more likely to encompass the fundamental niche of lynx, thus increasing the predictive performance of the model across the study area. In other words, while any one population is unlikely to represent the totality of a species' geographic distribution, a sufficient sample of multiple populations throughout a larger portion of its range is capable of describing individual populations quite well. Qiao et al. (2018) showed that SDMs were more transferable when more of the fundamental niche was used for model training, resulting in less extrapolation between calibration and transfer regions. Here, the covariates that had the most effect on lynx habitat capability were primarily temperature and moisture related, with the top four variables all related to snow, precipitation, or cold temperatures, as well as NDVI, a measure of long‐term forest presence or productivity. These results have conservation implications for the species' future at the southern range periphery under a changing climate, as temperature is likely to increase and snow to decrease if anthropogenic climate change continues unabated. Previous work has shown that warming trends are more severe in areas with mean annual temperatures in the range of 0°C to 5°C, due to a snow‐ice feedback loop where loss of snow causes lowered surface albedo, which in turn further speeds warming (Pepin & Lundquist, 2008). Our study area had a mean annual temperature ranging from −1°C to 12°C (Table A1), suggesting that snow‐ice feedback might influence warming patterns in lynx habitat, resulting in faster warming and decreased habitat suitability. King et al. (2020) found a similar susceptibility to changes in temperature and snow pack for the persistence of Canada lynx at their range periphery in Washington.

We found the amount of data provided by most GPS studies may greatly exceed what is necessary for peak SDM model performance and may be deleterious to model generalizability at some sizes, possibly reflected in the decreased transferability of our large dataset from the Montana population, as compared to the smaller dataset of Washington. Boria and Blois (2018) found that an SDM using approximately 13,000 occurrences from deer mice (*Peromyscus maniculatus*) decreased in predictive ability at large sample sizes, and that models with 10%–20% of the presence locations performed as well as those with greater percentages. Our results were similar, in that models with approximately 30% or more of our ~22,000 occurrences performed similarly. This number may be influenced by the number of individuals or sample size, however, as Wyoming, which had the fewest individuals and smallest sample, required closer to 70%–80% of the dataset to reach peak predictive performance. While the sample size of our Wyoming population was small compared to other datasets in our study, the number of presences was large (*n* = 670) compared to what is often recommended as the minimum sample size necessary for species distribution modeling (*n* ≈ 25, Hernandez et al., 2006; 50 < *n *< 100, Stockwell & Peterson, 2002). The Wyoming model performed well when assessed within the model training area, but exhibited poor transferability, which reinforces the need for caution in extrapolating even models that validate highly to novel areas. An aspect of GPS data collection that we acknowledge we were unable to address here was the effect of fix rate on GPS data efficiency. The fix rate, which determines the number of GPS locations taken during a given time period, was similar for GPS data from all three study populations, with one fix per hour in Montana, one fix per four hours in Washington, and one fix per three hours in Wyoming. Previous work has shown that autocorrelation increases with increased fix rate (Fieberg et al., 2010); thus, when applying methods used here, a reduction to 30% of the data should be considered when fix rates are similar, while a further reduction in data will likely be necessary for datasets with faster fix rates and less reduction when fix rate is slower.

Sensitive carnivores require large‐scale monitoring to evaluate population status (Golding et al., 2018). These efforts are aided by SDMs that spatially map the likelihood of species presence or habitat suitability so ecologists and managers can evaluate management actions such as recreation or timber production (Rowland & Vojta, 2013). Our work here provides the most comprehensive evaluation of lynx habitat at the species' southern range periphery in the northwestern United States. In addition, we used an extensive sample of known lynx locations across the study area to evaluate model performance. As such, this SDM for lynx will be central to conservation planning across the northwestern United States. The map we generated provides users with consistent predictions across multiple jurisdictions, allowing land management decisions to be made and applied consistently over a broad area. The model delineated large areas of high‐quality contiguous lynx habitat in parts of the Rocky Mountains in western Montana and the Cascade Range in Washington and British Columbia. With the use of our regional model, we also predicted the probability and spatial distribution of habitat that lacked detailed GPS data. These smaller but still potentially suitable habitat patches were in areas outside of the three main populations, including portions of northern Idaho, the Kettle Mountains in Washington, and scattered areas in the Bitterroot and Pioneer Mountains in Montana. Although some habitat patches may be too small to support long‐term occupancy and reproduction, they may provide valuable areas of refuge or connectivity to maintain population persistence at the species' southern range periphery (Walpole et al., 2012). The delineation of habitat patches in Canada also provides important conservation information, since these areas often act as “source” populations for the lynx populations in the northwestern United States (Schwartz et al., 2002). The methods we used here should provide managers and conservationists with a more refined depiction of “high” probability habitat, allowing conservation actions, which are limited by time and resources, to be focused on areas which will be the most beneficial to lynx.

## CONFLICT OF INTEREST

The authors declare no conflict of interest.

## AUTHOR CONTRIBUTIONS

**Lucretia E. Olson:** Conceptualization (equal); formal analysis (lead); writing‐original draft (lead); writing‐review & editing (equal). **Nichole Bjornlie:** Conceptualization (supporting); data curation (equal); writing‐review & editing (equal). **Gary Hanvey:** Conceptualization (supporting); data curation (equal); writing‐review & editing (equal). **Joseph D. Holbrook:** Conceptualization (supporting); writing‐review & editing (equal). **Jacob S. Ivan:** Conceptualization (supporting); data curation (equal); writing‐review & editing (equal). **Scott Jackson:** Conceptualization (supporting); writing‐review & editing (equal). **Brian Kertson:** Conceptualization (supporting); data curation (equal); writing‐review & editing (equal). **Travis King:** Conceptualization (supporting); data curation (equal); writing‐review & editing (equal). **Michael Lucid:** Conceptualization (supporting); data curation (equal); writing‐review & editing (equal). **Dennis Murray:** Conceptualization (supporting); data curation (equal); writing‐review & editing (equal). **Robert Naney:** Conceptualization (supporting); data curation (equal); writing‐review & editing (equal). **John Rohrer:** Conceptualization (supporting); data curation (equal); writing‐review & editing (equal). **Arthur Scully:** Conceptualization (supporting); data curation (equal); writing‐review & editing (equal). **Daniel Thornton:** Conceptualization (supporting); Data curation (equal); writing‐review & editing (equal). **Zachary Walker:** Conceptualization (supporting); data curation (equal); writing‐review & editing (equal). **John R. Squires:** Conceptualization (equal); data curation (equal); writing‐review & editing (equal).

## Data Availability

The data that support the findings of this study are openly available in figshare at https://doi.org/10.6084/m9.figshare.13383023.

## APPENDIX A

Initially, we considered 41 environmental predictors: 24 related to climate, 3 related to soil conditions, 7 related to topography, 5 related to vegetation, and 2 depicting anthropogenic factors (Table A1). Since many of these covariates were highly correlated with each other, we initially ran a single global model with all covariates using only machine‐learning modeling methods (global boosted models, random forest, and multiple adaptive regression splines) since these are known to be robust to correlation among covariates (Li & Wang, 2013). We used the “biomod2” package to run models, and included a measure of variable importance, created by randomizing a single variable, making new model predictions, and comparing these predictions to predictions from the entire model. Predictions that were very similar indicate little importance of the randomized variable, whereas very different predictions indicate that the variable was an important contributor. We ran 10 model repetitions using different sets of pseudoabsences each time, ranked the variables by their importance at each repetition, and calculated the median rank for each variable across all 3 models and 10 repetitions. We then eliminated covariates with pairwise correlations of |*r*| > 0.7, keeping the higher‐ranked covariate in the pair. This resulted in a final covariate set of 12 topographic and climatic variables, 2 vegetation and 2 anthropogenic covariates.

**TABLE A1:** Table of 41 environmental predictors initially screened for use in species distribution models. The type of predictor (climate, soil, topography, vegetation, anthropogenic) is given in the “Category” column, as well as a description of the covariates, the units (if not unitless) and range of covariate values, the original source of the data, and whether the variable was used in the final covariate set

Category	Covariate description	Units; range	Source	Used
Climate	Degree days below 18°C	2,462–9,232	1	
Climate	Frost‐free period	days; 15–198	1	
Climate	Heat load	0.42–0.92	2	X
Climate	Integrated moisture index	60–6,055	2	X
Climate	Maximum snow density	0–0.45	3	
Climate	Mean annual precipitation	mm; 255–4,837	1	
Climate	Mean annual relative humidity	%; 44–75	1	X
Climate	Mean annual temperature	°C; −1 to 12	1	
Climate	Mean snow density	0–0.28	3	
Climate	Mean summer (May to Sep) precipitation	mm; 111–596	1	
Climate	Mean temperature in coldest month	°C; −9 to 2	1	X
Climate	Mean temperature in warmest month	°C; 9 to 24	1	
Climate	Minimum snow density	0–0.19	3	X
Climate	Number of frost‐free days	days; 28–277	1	
Climate	Precipitation as snow	mm; 7–1,463	1	
Climate	Snow density difference	0–0.29	3	
Climate	Snow depth	m; 0–3	3	
Climate	Snow water equivalent	m; 0–1.2	3	X
Climate	Summer heat moisture index (Mean Temp Warmest Mo/(Mean Summer Precip/1,000))	22–216	1	
Climate	Summer mean temperature (Jun to Aug)	°C; 8–23	1	
Climate	Summer precipitation (Jun to Aug)	mm; 56–305	1	X
Climate	Variation in snow density	0–0.05	3	
Climate	Winter mean temperature (Dec to Feb )	°C; −9 to 2.7	1	
Climate	Winter precipitation (Dec to Feb)	mm; 34–846	1	X
Soil	Soil bulk density at 5 cm (The lighter the bulk density then potentially more organic matter and better water holding capacity)	(kg/m^3^) 200–2,870	4	
Soil	Soil organic carbon at 5 cm	‰ (g/kg) 0–450	4	
Soil	Soil pH (The wetter the habitat in a general sense then the lower the ph. Alpine fir and that climatic zone would be expected to have a low pH from litter, high precipitation and cold temps)	pH × 10 20–110	4	X
Topography	Elevation	m, 0–5,089	5	
Topography	Roughness	unitless, 0–82,216	2	
Topography	Slope	degrees, 0–81	6	
Topography	Surface area	unitless, 1–5.5	7	X
Topography	3‐D surface area	square m; 62,500–346,263	7	
Topography	TPI (1k, 5k, 10k)	unitless, 10k: −1,000 to 1,100, 5k: −806 to 891, 1k: −350 to 430	8	X
Topography	Compound Topographic Index	2.3–23.7	2	X
Veg	Enhanced vegetation index	−1 to 1	5, 9	
Veg	Normalized burn ratio	−1 to 1	5, 9	
Veg	Normalized difference vegetation index	−1 to 1	5, 9	X
Veg	Forest heterogeneity (Standard deviation of forest presence or absence at 1k, 5k, 10k scales)	unitless; 1k: 0–47, 5k: 0–43, 10k: 0–43	6	X
Veg	Percent forest cover	%, 0–100	5, 10	
Anthro	Lights from cities, towns, and other sites with persistent lighting, including gas flares, as a proxy for human disturbance	unitless; 0–1,106	5	X
Anthro	Road density	km/km^2^; 0–50	11	X

Note

Data Sources:

1: Wang, T. et al. 2016. Locally downscaled and spatially customizable climate data for historical and future periods for North America. PLoS One 11: e0156720.

2: Evans, J. S. et al. 2014. An ArcGIS toolbox for surface gradient and geomorphometric modeling, version 2.0. https://evansmurphy.wixsite.com/evansspatial/arcgis‐gradient‐metrics‐toolbox, Accessed June 2017.

3: National Operational Hydrologic Remote Sensing Center 2004. Snow data assimilation system (SNODAS) data products at NSIDC, Version 1. https://nsidc.org/data/g02158, Accessed June 2017.

4: Hengl, T. et al. 2017. SoilGrids250m: Global gridded soil information based on machine learning. PLoS 12: e0169748.

5: Gorelick, N. et al. 2017. Google Earth Engine: Planetary‐scale geospatial analysis for everyone. Remote Sensing of the Environment 202:18–27.

6: ESRI 2011. ArcGIS Desktop: Release 10.5. Redlands, CA: Environmental Systems Research Institute.

7: Jenness, J. 2013. DEM Surface Tools for ArcGIS. ‐Jenness Enterprises. http://www.jennessent.com/arcgis/surface_area.htm, Accessed June 2017.

8: Jenness, J. et al. 2013. Land Facet Corridor Designer: Extension for ArcGIS. ‐ Jenness Enterprises. http://www.jennessent.com/arcgis/land_facets.htm, Accessed June 2017.

9: Landsat 5 and 8, United States Geological Survey Data, 2000 – 2015. https://glovis.usgs.gov/, Accessed June 2017.

10: Hansen, M. C. et al. 2013. High‐Resolution Global Maps of 21st‐Century Forest Cover Change. Science 342:850–853.

11: OpenStreetMap Foundation 2017. OpenStreetMap. https://www.openstreetmap.org/about, Accessed June 2017.

## APPENDIX B

Pairwise correlations between each of the 16 covariates used in the final species distribution model. Covariate pairs correlated at *r* > |0.6| are shown in bold.

**Table:**

	Heat Load	Int Moist	Temp Cold Mo	Snow Den	NDVI	Lights	Soil pH	Sum Prec	Win Prec	Rel Hum	Road Den	Surf Area	Snow Water Eq	TPI	Forest Het
Comp Topo Index	−0.07	0.34	0.08	−0.20	0.02	0.10	0.26	−0.20	−0.20	−0.23	0.19	−0.42	−0.21	−0.36	−0.17
Heat Load	1.00	−0.01	0.01	0.02	0.05	−0.01	−0.03	0.02	0.03	0.01	−0.01	0.07	0.02	0.02	0.03
Int Moisture		1.00	0.01	−0.04	0.00	0.01	0.08	−0.04	−0.04	−0.05	0.03	−0.05	−0.03	−0.10	−0.05
Temp Cold Mo			1.00	−0.15	0.32	0.13	0.13	−0.39	0.22	0.12	0.33	−0.15	−0.33	−0.23	−0.28
Snow Density				1.00	0.33	−0.10	**−0.72**	0.31	0.57	0.51	−0.17	0.31	**0.68**	0.12	0.25
NDVI					1.00	−0.05	−0.46	0.10	0.31	0.32	0.09	−0.09	0.11	−0.15	0.02
Lights						1.00	0.14	−0.09	−0.08	0.00	0.53	−0.09	−0.12	−0.06	−0.06
Soil pH							1.00	−0.51	**−0.65**	−0.59	0.22	−0.31	**−0.66**	−0.30	−0.36
Summer Precip								1.00	0.38	0.45	−0.24	0.39	0.41	0.25	0.21
Winter Precip									1.00	0.51	−0.09	0.37	**0.61**	0.19	0.10
Relative Humid										1.00	−0.11	0.35	0.38	0.27	0.16
Road Density											1.00	−0.26	−0.29	−0.21	−0.15
Surface Area												1.00	0.37	0.17	0.28
Snow Water Eq													1.00	0.22	0.27
TPI														1.00	0.08
Forest Het															1.00

Abbreviations: Comp Topo Index, Compound Topographic Index; Forest Het, Forest Heterogeneity; Int Moisture, Integrated Moisture; Lights, Night Lights; NDVI, Normalized Difference Vegetation Index; Rel Hum, Relative Humidity; Road Den, Road Density; Snow Den, Snow Density; Snow Water Eq, Snow Water Equivalent; Sum Prec, Summer Precipitation; Surf Area, Surface Area; Temp Cold Mo, Mean Temperature in the Coldest Month; TPI, Topographic Position Index; Win Prec, Winter Precipitation.

## APPENDIX C

**TABLE C1:** The eigenvalue, a measure of the amount of variation retained by each principal component, percent variance contribution, and cumulative percent variance contribution of each dimension in the principal components analysis (PCA)

	Eigenvalue	Percent variance	Cumulative percent variance
Dim.1	3.37	21.06	21.06
Dim.2	1.90	11.85	32.92
Dim.3	1.56	9.75	42.67
Dim.4	1.52	9.51	52.18
Dim.5	1.33	8.31	60.48
Dim.6	0.99	6.22	66.70
Dim.7	0.96	6.02	72.72
Dim.8	0.93	5.82	78.54
Dim.9	0.77	4.79	83.33
Dim.10	0.64	4.03	87.35
Dim.11	0.60	3.72	91.08
Dim.12	0.46	2.88	93.96
Dim.13	0.32	2.01	95.97
Dim.14	0.30	1.85	97.82
Dim.15	0.20	1.27	99.09
Dim.16	0.15	0.91	100.00

Note

The first two PCA axes explain 32.92% of the variance in the covariates.

**TABLE C2:** The percent contribution of each covariate to the first five principal component dimensions

	Dim.1	Dim.2	Dim.3	Dim.4	Dim.5
Compound Topographic Index	6.11	6.56	12.91	14.69	0.24
Heat Load	1.55	0.01	1.86	2.83	1.55
Integrated Moisture	2.07	4.17	16.43	11.30	0.79
Mean Temp in Coldest Month	0.46	7.28	2.34	2.74	39.24
Snow Density	5.00	0.34	0.61	24.69	5.67
Normalized Difference Veg Index	0.30	28.65	2.15	2.39	2.80
Night Lights	0.65	0.31	0.68	3.50	0.56
Soil pH	16.89	0.36	0.14	7.78	5.50
Summer Precipitation	6.05	10.51	0.05	2.16	23.51
Winter Precipitation	17.32	3.84	6.69	1.40	1.86
Relative Humidity	8.08	0.00	10.28	15.53	3.68
Road Density	1.97	13.34	1.69	0.43	8.83
Surface Area	10.05	0.97	2.89	9.24	2.74
Snow Water Equivalent	15.94	0.56	15.55	0.21	0.00
Topographic Position Index	6.96	6.87	12.69	0.92	0.27
Forest Heterogeneity	0.61	16.20	13.03	0.20	2.78

Note

Dimension 1 is dominated by moisture‐related covariates including summer and winter precipitation, soil pH, and relative humidity, while dimension 2 is dominated by forest‐related covariates including long‐term NDVI, forest heterogeneity, and road density.

## APPENDIX D

**TABLE D1:** Model validation, as measured with continuous Boyce Index, for all species distribution models generated for Canada lynx in the northwestern United States

Validation data source	Data location	Model being tested	Background	Performance in			
				MT	WA	WY	Region
Withheld	Region	Unequal	Region	1.000^a^	1.000^a^	0.996^a^	0.998^a^
		WA equal	Region	1.000^a^	0.985	0.992^b^	0.992^b^
		WY equal	Region	1.000^a^	0.943	0.985	0.985
	Population	MT	Region	1.000^a^	−0.811	0.220	0.481
		MT	Population	1.000^a^	−0.258	−0.201	0.468
		WA	Region	0.919^b^	0.998	0.561	0.774
		WA	Population	0.697	0.999^b^	0.953	0.998^a^
		WY	Region	−0.808	0.897	0.954	0.498
		WY	Population	−0.182	0.138	0.973	0.897

				MT	WA	WY	Region
Independent	Region	Unequal	Region	0.8670	0.9190^b^	0.9020	0.9600^b^
		WA equal	Region	0.9860^b^	0.8870	0.9070^b^	0.9450
		WY equal	Region	0.9880^a^	0.9200^a^	0.9610^a^	0.9660^a^
	Population	MT	Region	0.9240	0.6270	0.5690	0.8130
		MT	Population	0.8710	0.6390	0.8140	0.6210
		WA	Region	0.3250	0.9020	0.3580	0.7600
		WA	Population	0.6520	0.8940	0.8870	0.9560
		WY	Region	0.0150	0.7680	0.4960	0.4500
		WY	Population	−0.3240	0.3920	0.7160	0.8030

Note

Values in each column marked with a superscript “a” indicate best model performance in that population, superscript “b” indicate second best.

**TABLE D2:** Model validation, as measured with minimum predicted area at 90% threshold, for all species distribution models generated for Canada lynx in the northwestern United States

Validation data source	Data location	Model being tested	Background	Performance in			
				MT	WA	WY	Region
Withheld	Region	Unequal	Region	17,290^b^	13,092	21,042	83,267
		WA equal	Region	20,647	8,570^b^	8,463^a^	40,790^a^
		WY equal	Region	39,667	13,816	17,585	64,957^b^
	Population	MT	Region	22,411	25,538	64,727	297,087
		MT	Population	14,112^a^	26,188	63,155	235,920
		WA	Region	182,116	11,178	50,266	327,534
		WA	Population	132,787	7,962^a^	43,122	159,878
		WY	Region	212,295	30,605	12,224	307,298
		WY	Population	203,309	58,771	11,395^b^	280,977

				MT	WA	WY	Region
Independent	Region	Unequal	Region	210,714	22,954	22,331^a^	203,419^b^
		WA equal	Region	205,783	21,802^b^	24,842	213,308
		WY equal	Region	207,134	25,685	24,388^b^	199,545^a^
	Population	MT	Region	150,449^a^	30,488	44,899	254,118
		MT	Population	181,272^b^	27,512	47,534	228,531
		WA	Region	217,707	17,961^a^	50,266	348,497
		WA	Population	213,574	24,580	24,877	263,568
		WY	Region	254,859	36,697	52,116	346,885
		WY	Population	243,840	48,045	39,358	283,051

Note

Values are given in km^2^, indicating the minimum area required to correctly identify 90% of Canada lynx locations present in a presence/absence categorical map. Lower values indicate greater model efficiency (less area for the same amount of error). Values in each column marked with a superscript “a” indicate best model performance in that population, superscript “b” indicate second best.
